# Heart–Lung Interactions in Pulmonary Hypertension due to Heart Failure With Preserved Ejection Fraction

**DOI:** 10.1002/cph4.70168

**Published:** 2026-05-15

**Authors:** Pratima Prabala, Farhan Raza, Naomi C. Chesler

**Affiliations:** ^1^ Edwards Lifesciences Foundation Cardiovascular Innovation and Research Center and Department of Biomedical Engineering University of California Irvine California USA; ^2^ Department of Medicine, Cardiovascular Division University of Wisconsin‐Madison Madison Wisconsin USA

**Keywords:** cardiopulmonary physiology, heart–lung interactions, molecular and cellular crosstalk, pulmonary vascular disease, pulmonary vascular resistance and compliance, pulsatile afterload, right ventricular‐pulmonary vascular coupling

## Abstract

Pulmonary hypertension associated with heart failure with preserved ejection fraction (PH‐HFpEF) is a common and high‐risk clinical condition characterized by the convergence of pulmonary vascular dysfunction and right ventricular (RV) stress. Despite its prevalence, the pathophysiological mechanisms of development and progression of PH‐HFpEF remain largely unknown. Consequently, there are no current therapies that target PH‐HFpEF other than optimization of treatment for HFpEF and some limited adaptation of therapies for pulmonary artery hypertension (PAH). This review examines PH‐HFpEF through the lens of heart–lung interactions, integrating evidence across hemodynamic, molecular, and cellular scales. We highlight how chronic venous pressure overload, impaired pulmonary vascular reserve, and increased pulsatile load reshape RV afterload and contribute to RV‐pulmonary arterial uncoupling, particularly during physiological stress such as exercise. We further discuss how endothelial dysfunction, context‐dependent signaling pathways, and systemic comorbidities modify pulmonary vascular remodeling and RV adaptation in ways that differ from PAH. Emerging roles for extracellular vesicles and other circulating mediators as vehicles of cardiopulmonary signaling are considered, alongside current experimental and clinical tools for studying these processes. By examining physiological and molecular insights in parallel, this review identifies key gaps in disease modeling, phenotyping, and mechanistic understanding, while emphasizing the need for approaches that link cardiopulmonary function to underlying biology. Such strategies are essential for advancing disease phenotyping and developing targeted therapies for PH‐HFpEF.

## Graphical abstract

This review considers heart–lung interactions in pulmonary hypertension due to heart failure with preserved ejection fraction across scales, including organ, tissue, and cell scales. Key knowledge gaps in preclinical disease models, tools for clinical phenotyping, and underlying biological mechanisms are identified for future studies.

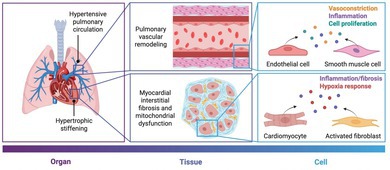

## 1 Introduction

Effective cardiopulmonary function depends on tight coordination between the right ventricle (RV) and the pulmonary vasculature, which together regulate blood flow, pressure, and gas exchange across the lungs. Under physiological conditions, this coupling between the RV and pulmonary vasculature ensures optimal matching of flow and pressure, preserving hemodynamic stability and oxygen delivery (Haddad et al. 2008; Vonk Noordegraaf et al. 2017). In heart failure with preserved ejection fraction (HFpEF), this balance is disrupted by the development of pulmonary hypertension (PH), a common complication that places chronic pressure overload on the RV. PH‐HFpEF represents one of the most common forms of PH worldwide, affecting approximately 50%–80% of patients with HFpEF, and is a major determinant of exercise intolerance, RV dysfunction, and mortality (Lam et al. 2009; Salamon et al. 2014; Ameri et al. 2024).

HFpEF comprises a heterogenous group of overlapping pathophysiological mechanisms (Vachiéry et al. 2019). The most common pathobiological abnormality in HFpEF is adverse left ventricular remodeling, in which increased myocardial stiffness and concentric hypertrophy result in elevated intracardiac pressures and secondary PH. In an aging population with a growing burden of atrial fibrillation, left atrial myopathy, which is characterized by atrial dilation, impaired compliance, and loss of reservoir function, may represent a dominant mechanism even in the absence of severe ventricular pathology (Patel and Shah 2020). In HFpEF, chronically elevated left atrial pressure drives pulmonary vascular remodeling, with endothelial dysfunction and metabolic‐inflammatory signaling acting as key modifiers of disease progression. These stressors remodel both the pulmonary veins and arteries, resulting in progressive vascular stiffening. Consequently, the RV is exposed to an increased hemodynamic load that extends beyond elevations in mean pulmonary pressure (Al‐Omary et al. 2020). Exaggerated ventricular interdependence due to pericardial restraint constitutes another important contributor, whereby RV dilation and elevated pericardial pressures mechanically constrain left ventricular filling, leading to elevated intracardiac pressures despite reduced LV volumes (Borlaug and Reddy 2019; Zampierollo‐Jaramillo et al. 2024). In the elderly, aging‐associated HFpEF can be superimposed on established precapillary pulmonary arterial hypertension (PAH), such as in connective tissue disease, creating a distinct phenotype with unique diagnostic and therapeutic challenges. Once both postcapillary and precapillary PH are present, the RV is forced to adapt to a complex hemodynamic environment that includes increased pulsatile load and reduced pulmonary vascular reserve. Failure of the RV adaptive response leads to RV‐pulmonary arterial (RV‐PA) uncoupling, a critical transition associated with worsening symptoms and adverse clinical outcomes in PH‐HFpEF (Borlaug et al. 2016). Collectively, the heterogeneity of PH‐HFpEF complicates diagnosis and management, often necessitating physiologic stress testing, including exercise during invasive hemodynamic assessments, to unmask abnormal cardiopulmonary responses and prevent inappropriate treatment (Rothbard et al. 2018; Maron et al. 2024).

Traditionally, heart–lung interactions in PH‐HFpEF were examined through mechanical and hemodynamic parameters, including pulmonary vascular resistance, compliance, and RV‐PA coupling (Vonk Noordegraaf et al. 2017; Naeije and Manes 2014). More recent evidence indicates that cardiopulmonary communication in this condition extends into molecular, cellular, and metabolic signaling pathways that mediate bidirectional crosstalk between the pulmonary vasculature and heart (Lahm et al. 2025; Hemnes et al. 2024). Inflammatory signaling, endothelial dysfunction, sex hormone imbalance, metabolic stress, and extracellular vesicle‐mediated communication have all emerged as critical modulators of pulmonary vascular remodeling and RV function in HFpEF‐associated PH (Ventetuolo et al. 2015; Obokata, Reddy, et al. 2017; Buffolo et al. 2022).

**FIGURE 1:**
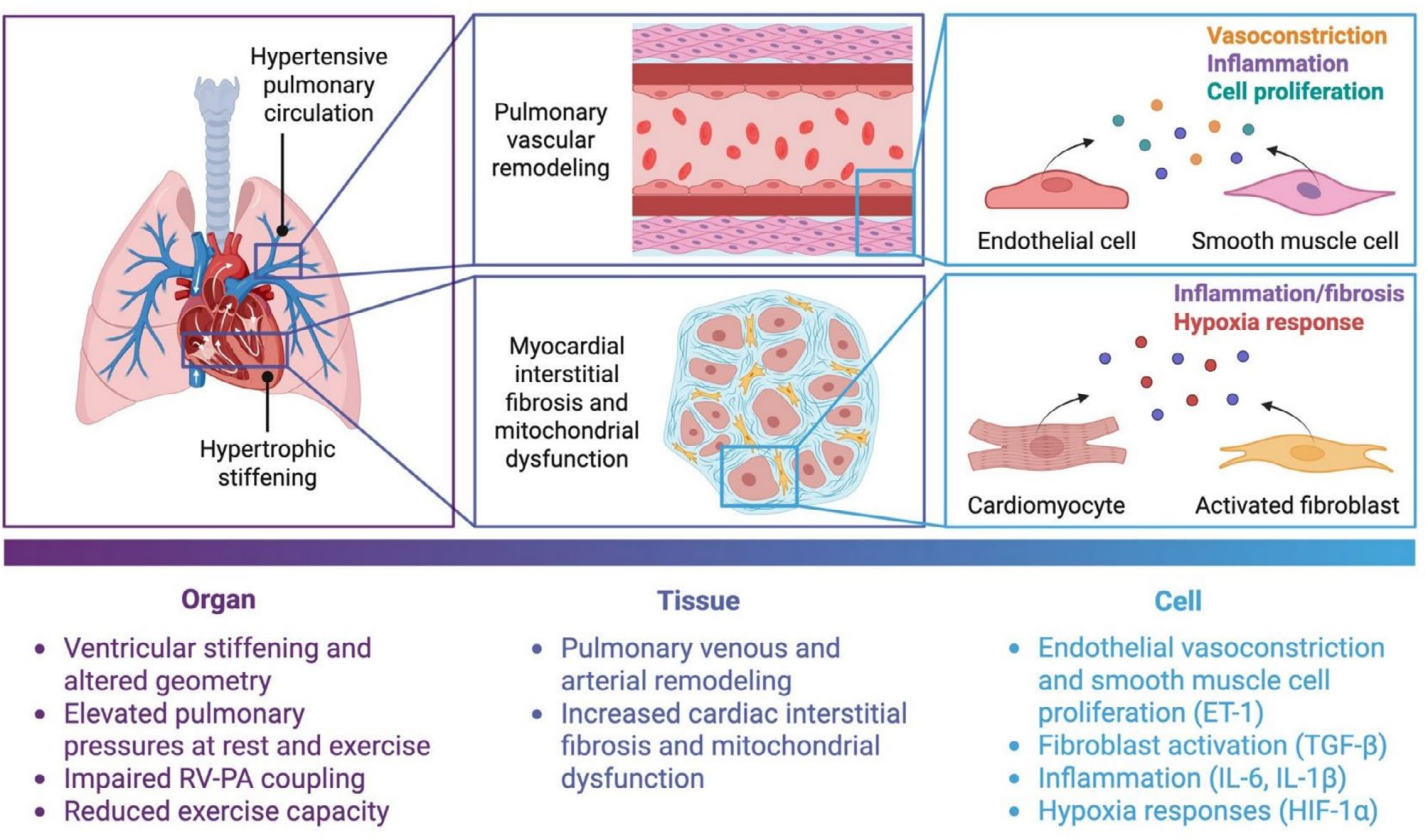
Multiscale heart–lung interactions underlying PH‐HFpEF.

In this review, we focus on PH‐HFpEF as a paradigmatic example of disrupted heart–lung interactions at multiple scales (Figure 1). We first summarize current understanding of RV‐pulmonary vascular hemodynamic coupling in PH‐HFpEF, emphasizing the roles of resistance, compliance, and pulsatile load. We then discuss emerging insights into molecular and extracellular signaling mechanisms that link pulmonary vascular dysfunction to RV remodeling in this condition. Finally, we discuss experimental and translational approaches to study cardiopulmonary interactions in PH‐HFpEF and highlight key knowledge gaps that must be addressed to enable mechanism‐based, personalized therapies for this rapidly growing patient population.

## 2 Hemodynamic Coupling Between the Heart and Lung in PH‐HFpEF

In PH‐HFpEF, heart–lung interactions are defined by a distinct pattern of hemodynamic coupling between the RV and pulmonary circulation. Unlike PAH, where RV afterload arises primarily from precapillary vascular remodeling, PH‐HFpEF develops in the setting of chronically elevated left atrial pressure (Csósza et al. 2022), which is transmitted to the pulmonary veins and capillaries and initiates biomechanically driven pulmonary venous and capillary remodeling that progressively extends into the arterial compartment (Allen et al. 2023). As a result, the RV is exposed to a mixed postcapillary‐precapillary load that is dynamic, heterogeneous, and highly sensitive to changes in blood volume and exercise (Borlaug et al. 2016). Importantly, pulmonary vascular remodeling in PH‐HFpEF may not be solely maladaptive. In the setting of chronically elevated left‐sided filling pressures, increases in precapillary resistance and vascular stiffening have been proposed as compensatory responses that limit transmission of pressure to the pulmonary capillary bed, thereby protecting against overt cardiogenic pulmonary edema. However, this adaptive response occurs at the expense of increased RV afterload, ultimately contributing to RV dysfunction and RV–pulmonary arterial uncoupling as disease progresses.

A defining feature of PH‐HFpEF is early loss of pulmonary vascular compliance, often occurring before elevations in pulmonary vascular resistance (PVR). Postcapillary pulmonary hypertension, reflected by elevated pulmonary capillary wedge pressure, impairs the normal distension and recruitment of pulmonary microvasculature during increased flow, thereby amplifying pulsatility and RV load (Tedford et al. 2012). Consequently, even modest increases in cardiac output, such as during exercise, produce disproportionate rises in pulmonary artery pressure. This impaired pulmonary vascular reserve exposes the RV to excessive pulsatile load during activities of daily living, a component of afterload that is not adequately captured by resting mean pulmonary pressures. Thus, in PH‐HFpEF, RV stress is frequently underestimated when assessed using traditional pressure‐based measurements obtained during rest alone.

The interaction between steady and pulsatile components of RV afterload is particularly relevant in PH‐HFpEF. While PVR reflects resistance to mean flow, pulmonary arterial compliance governs the buffering of systolic ejection and the timing of wave reflections. In PH‐HFpEF, arterial stiffening accelerates the return of reflected pressure waves into systole, increasing RV wall stress and myocardial oxygen demand (Su et al. 2016). This hemodynamic environment contrasts with PAH, where resistance dominates early disease and helps explain why RV dysfunction can occur in PH‐HFpEF despite relatively modest elevations in resting PVR (Wezenbeek et al. 2021).

Hemodynamic RV‐pulmonary arterial (PA) coupling offers a useful way to understand how the RV adapts to increased load in PH‐HFpEF, although its interpretation must account for disease‐specific features (Vanderpool et al. 2015). In PH‐HFpEF, effective arterial elastance (Ea) rises not only because of increased vascular resistance but also due to reduced pulmonary vascular compliance (Thenappan et al. 2016). Early in the disease course, the RV can partially compensate at rest by undergoing concentric hypertrophy and increasing contractility (Ees), thereby preserving coupling. However, the ability of the RV to increase contractile performance during stress is often limited in HFpEF by reduced contractile reserve, metabolic dysfunction, and common comorbidities such as obesity, diabetes, and systemic inflammation (Borlaug et al. 2016; Packer 2025). As these constraints persist, RV‐PA uncoupling emerges, most clearly during exercise, and is associated with exercise intolerance, diminished quality of life, and increased rates of heart failure hospitalization.

**FIGURE 2:**
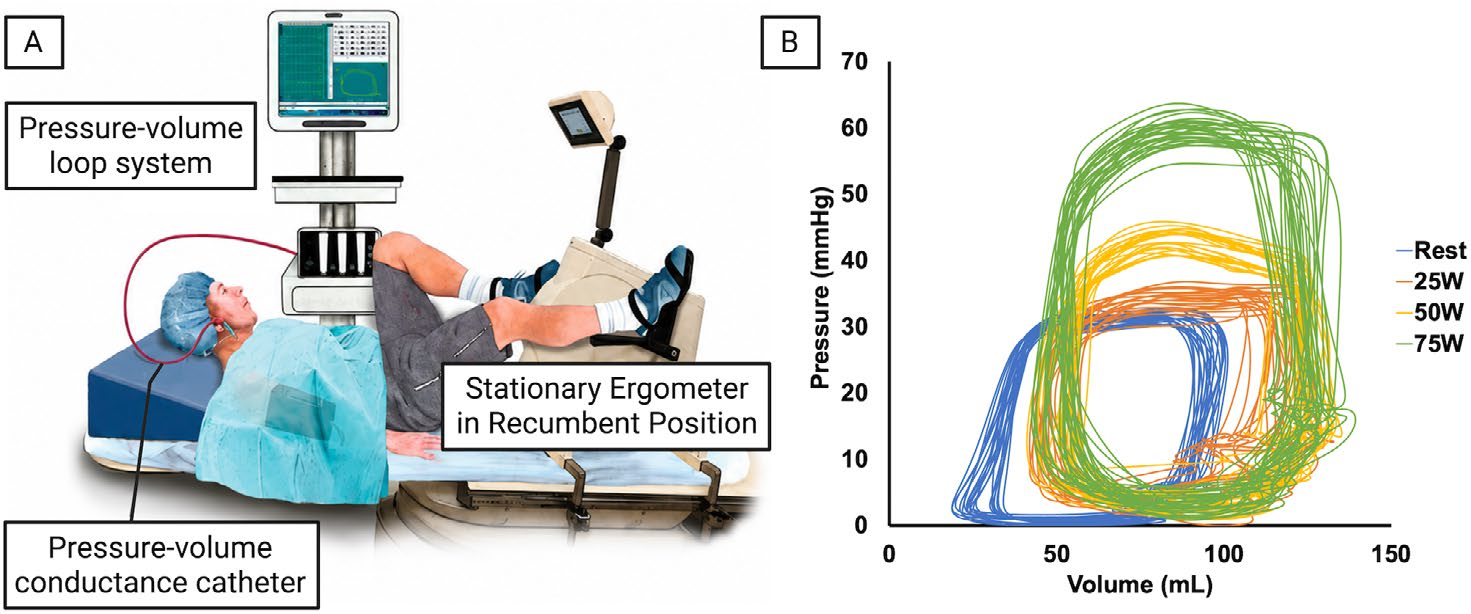
Invasive exercise RV pressure‐volume analysis. (A) Experimental setup for pressure‐volume loop acquisition during supine exercise (adapted from Raza et al. 2022). (B) Exercise‐induced changes in RV pressure‐volume loops in PH‐HFpEF (raw data from Raza et al. 2022).

Hemodynamic assessment of PH‐HFpEF therefore requires approaches that extend beyond resting measurements. Pressure‐volume analysis, exercise catheterization, and wave reflection metrics have demonstrated that RV dysfunction in PH‐HFpEF is driven by a combination of elevated filling pressures, impaired compliance, and exaggerated pulsatile load (Obokata, Reddy, et al. 2017; Lechuga et al. 2025a). Consistent with this concept, patient‐derived RV pressure‐volume loops obtained during invasive exercise testing using approaches described in Raza et al. demonstrate marked rightward and upward shifts with increasing workload (Raza et al. 2022). These shifts reflect disproportionate increases in RV systolic pressure, limited stroke volume augmentation, and rising energetic demand despite relatively modest abnormalities at rest (Figure 2). Detailed descriptions of catheterization, exercise testing, and pressure‐volume loop analysis can be found in the Methods section (Raza et al. 2022). These exercise‐induced alterations provide direct physiological evidence of dynamic RV‐PA uncoupling in PH‐HFpEF. Emerging imaging techniques, including 4D flow magnetic resonance imaging (MRI) and echocardiographic measures of RV strain, are beginning to capture these dynamic features in vivo (Barker et al. 2014; Melenovsky et al. 2014). Together, these findings highlight PH‐HFpEF as a disorder of impaired heart–lung coupling, in which pulmonary vascular stiffening and loss of flow reserve increase RV load in ways that are not captured by resting pressure measurements alone.

## 3 Intercellular Communication Across the Cardiopulmonary Axis in PH‐HFpEF

### 3.1 Cellular Crosstalk That Drives Endothelial and Mitochondrial Dysfunction and Inflammation

Beyond altered hemodynamics, PH‐HFpEF is increasingly recognized as a biologically active cardiopulmonary disorder shaped by molecular and cellular signaling between and within the lungs and RV (Al‐Omary et al. 2020). Given the limited availability of mechanistic studies specifically in PH‐HFpEF, current understanding of molecular and cellular pathways is informed in part by studies in related conditions, including PAH and systemic HFpEF. These studies provide important biological insight, although their applicability to PH‐HFpEF should be interpreted within the context of disease‐specific differences. Chronically elevated left‐sided filling pressures, systemic inflammation, metabolic dysfunction, and endothelial injury create a signaling environment that is distinct from PAH (Rosenkranz et al. 2016). In this setting, pulmonary vascular remodeling arises from sustained pulmonary venous and capillary pressure overload, reduced endothelial nitric oxide bioavailability, and an impaired ability of the pulmonary circulation to dilate and recruit vessels during increased flow, rather than from primary precapillary obstruction. Invasive hemodynamic and exercise studies demonstrate that pulmonary vascular abnormalities in HFpEF are dynamic and heterogeneous, implicating endothelial dysfunction and impaired vasodilatory signaling as early contributors to disease progression rather than passive consequences of pressure overload (Borlaug et al. 2016). Together, these observations motivate a shift toward understanding PH‐HFpEF as a disorder of maladaptive interorgan signaling superimposed on mechanical stress.

Several paracrine mediators are central to heart–lung communication in PH‐HFpEF. Endothelin‐1 (ET‐1), produced by pulmonary endothelial and smooth muscle cells, is a potent vasoconstrictor and driver of pulmonary vascular smooth muscle proliferation and remodeling (Davenport et al. 2016). In PAH, ET‐1 signaling is considered causal, based on experimental evidence and clinical efficacy of endothelin receptor antagonists which improve hemodynamics and clinical outcomes in PAH patients. In contrast, while circulating ET‐1 levels are elevated in PH‐HFpEF patients and correlate with severity of PH and adverse outcomes, this association does not establish ET‐1 as a dominant disease driver in PH‐HFpEF. Animal studies in pressure‐overload and metabolic models relevant to HFpEF demonstrate that ET‐1 signaling promotes pulmonary vascular smooth muscle proliferation, and adverse myocardial remodeling (Kim et al. 2014; Duangrat et al. 2023). However, randomized trials of endothelin receptor antagonists in HFpEF have failed to improve clinical outcomes, indicating that while ET‐1 contributes to disease biology, it is unlikely to be a dominant or isolated driver in PH‐HFpEF (Liu et al. 2013; Packer et al. 2005). These findings underscore that signaling pathways with causal roles in PAH may exert more context‐dependent effects in PH‐HFpEF, in which pulmonary arterial remodeling occurs as a consequence of chronic venous pressure overload.

Endothelial nitric oxide (NO)‐cyclic guanosine monophosphate (cGMP) signaling provides an example of a molecular pathway whose impairment appears fundamental to the pathobiology of PH‐HFpEF. Under normal conditions, endothelial‐derived NO promotes pulmonary vasodilation, preserves vascular compliance, and enables recruitment of the pulmonary microvasculature during increases in blood flow. Early invasive cardiopulmonary exercise studies revealed that this adaptive vasodilatory response is diminished in HFpEF, particularly during exercise, when demands on the pulmonary circulation are highest. In a study using combined exercise catheterization and gas exchange measurements, Borlaug et al. demonstrated that HFpEF patients fail to appropriately reduce PVR or augment pulmonary vascular compliance as cardiac output rises, despite only modest elevations in resting pressures, implicating endothelial dysfunction and impaired NO bioavailability rather than fixed anatomic obstruction as the primary limitation on flow accommodation (Borlaug et al. 2010). Subsequent invasive exercise studies extended these findings, showing that patients with PH‐HFpEF exhibit exaggerated pulmonary pressure responses and impaired vascular reserve during exertion, further supporting a dynamic, signal‐driven defect in pulmonary vasodilation (Obokata, Kane, et al. 2017, Borlaug et al. 2016). Because NO‐cGMP signaling is a central regulator of pulmonary vasodilation and arterial compliance, these physiological observations provided a strong mechanistic rationale for targeting this pathway therapeutically. This rationale motivated clinical trials of agents designed to augment downstream NO signaling, including phosphodiesterase‐5 (PDE5) inhibitors, which prevent cGMP degradation, and soluble guanylate cyclase (sGC) stimulators, which enhance cGMP production independent of NO availability. However, large randomized trials of PDE5 inhibition in unselected HFpEF populations failed to improve exercise capacity or clinical outcomes, indicating that non‐targeted, systemic enhancement of NO‐cGMP signaling is ineffective across the heterogeneous HFpEF population (Redfield et al. 2013).

Hypoxia‐responsive and inflammatory signaling pathways further integrate pulmonary vascular and RV dysfunction in PH‐HFpEF. In HFpEF, activation of hypoxia‐responsive signaling pathways reflects localized vascular stress and endothelial dysfunction rather than serving as a primary initiating stimulus. Experimental studies demonstrate that endothelial activation of hypoxia‐inducible factor (HIF)‐dependent transcriptional pathways promote vasoconstrictor production, metabolic reprogramming, and pro‐inflammatory gene expression (Tang et al. 2017; Yu et al. 1999). Together, these processes contribute to pulmonary vascular remodeling and stiffening, thereby limiting pulmonary vascular compliance and vasodilatory reserve. In parallel, HFpEF is associated with heightened inflammatory signaling, with circulating cytokines such as IL‐6 and IL‐1β linked to worsened pulmonary hemodynamics and RV dysfunction (Alogna et al. 2023; Mooney et al. 2023; Engel Sällberg et al. 2023). Collectively, these pathways modulate disease severity by amplifying pulmonary vascular stiffness and increasing RV sensitivity to load.

Beyond hypoxia responses, accumulating evidence implicates mitochondrial dysfunction as a key metabolic consequence that links vascular stress to impaired cardiopulmonary adaptation in PH‐HFpEF. Mitochondrial dysfunction contributes to pulmonary vascular and RV remodeling in PH‐HFpEF by limiting cellular energy production under chronic pressure overload. Evidence from PAH models and studies of systemic vascular and cardiac cells in HFpEF suggests that mitochondria in endothelial cells, smooth muscle cells, and cardiomyocytes exhibit reduced capacity for oxidative phosphorylation and fatty acid oxidation, with a compensatory shift toward less efficient glycolytic metabolism (Sutendra and Michelakis 2014; Kumar et al. 2019). These changes lower cellular ATP availability and increase oxidative stress. In the pulmonary circulation, excess mitochondrial reactive oxygen species impair endothelial signaling, reduce NO production, and promote smooth muscle cell proliferation, accelerating vascular stiffening and luminal narrowing (Ryan et al. 2015; Sutendra and Michelakis 2014). In the RV, impaired mitochondrial energy production limits the ability to meet rising energetic demands during increased afterload, contributing to reduced contractile reserve and greater sensitivity to pulsatile stress during exercise (Müller et al. 2023). Mitochondrial dysfunction also interacts with hypoxia‐responsive and inflammatory signaling pathways, further promoting vascular remodeling and constraining adaptive RV responses.

Sex hormone signaling adds a critical layer to cardiopulmonary communication in PH‐HFpEF. Post‐menopausal women are disproportionately affected by HFpEF and PH‐HFpEF, and experimental and clinical evidence suggests that estrogen signaling supports endothelial resilience, limits fibrosis, and preserves RV diastolic function under pressure overload (Mendelsohn and Karas 1999). Animal studies demonstrate that intact estrogen signaling improves RV adaptation, whereas estrogen loss accelerates maladaptive remodeling (Umar et al. 2011). These findings parallel clinical observations that pre‐menopausal women with PH often maintain better RV function despite similar or greater pulmonary vascular burden when compared with men, underscoring sex‐specific signaling as an important modifier of heart–lung interactions in PH‐HFpEF (Ma et al. 2025).

**FIGURE 3:**
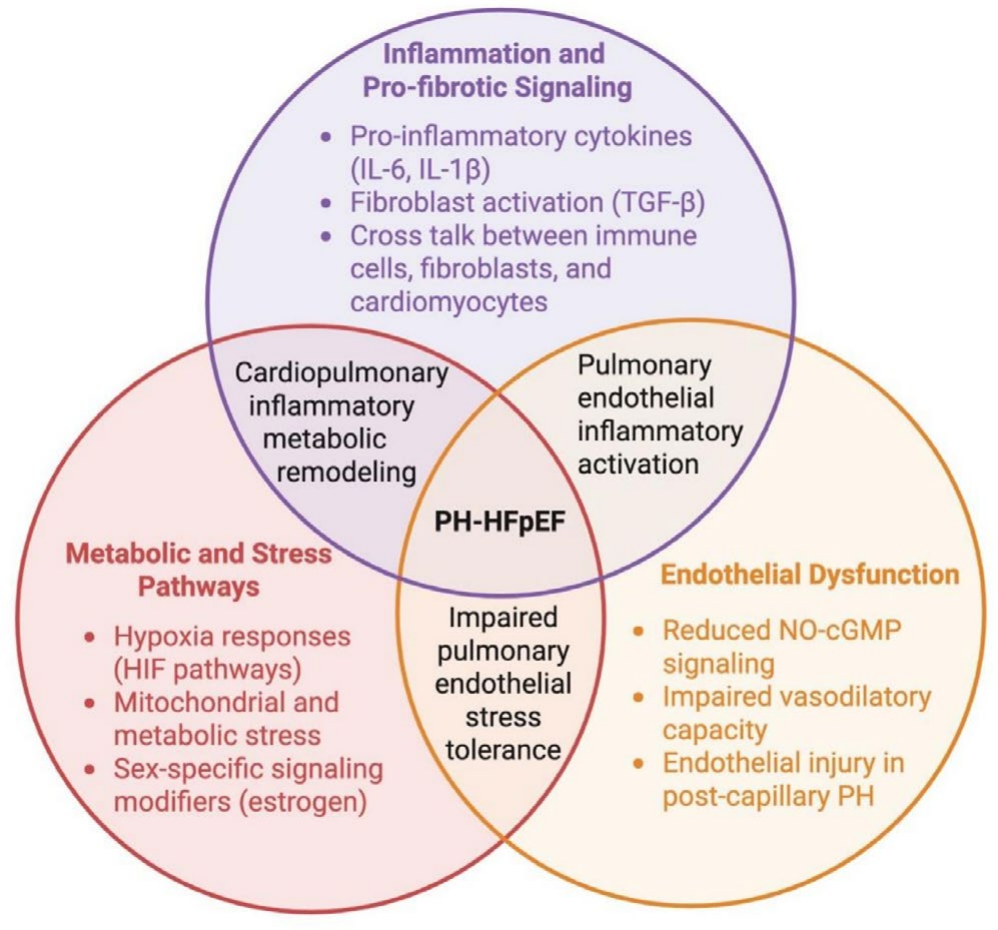
Convergent inflammatory, metabolic, and endothelial signaling mechanisms in PH‐HFpEF.

Together, the inflammatory, metabolic, endothelial, hypoxia‐responsive, and sex hormone‐dependent pathways described above converge to shape pulmonary vascular remodeling and RV adaptation in PH‐HFpEF. These interacting mechanisms are summarized in Figure 3, which highlights how shared signaling processes across the cardiopulmonary unit amplify vascular stiffening and RV dysfunction.

### 3.2 Circulating Mediators That Drive Multiscale Dysfunction

In addition to soluble cytokines and growth factors, emerging evidence suggests that communication between the pulmonary vasculature and RV in PH‐HFpEF is mediated in part by extracellular vesicles (EVs). EVs are membrane‐bound nanoparticles, including exosomes and microvesicles, that transport bioactive cargo such as microRNAs (miRNAs), proteins, and lipids between cells, enabling coordinated responses across distant tissues. Pulmonary endothelial cells, smooth muscle cells, fibroblasts, and immune cells release EVs in response to mechanical stress, hypoxia, and inflammation, conditions that are prominent in PH‐HFpEF. Importantly, EVs are stable in the circulation and capable of modifying recipient cell behavior, making them well suited to act as long‐range messengers. While there is limited literature dedicated to the role of EVs in PH‐HFpEF, some reviews have focused on EVs as plausible “hidden players” linking common HFpEF comorbidities, such as obesity, diabetes, and vascular inflammation, to endothelial dysfunction and downstream remodeling (Gohar et al. 2018). An overview of microRNAs with established roles in pulmonary vascular and cardiac remodeling relevant to PH‐HFpEF is provided in Table 1. These include miRNAs that promote smooth muscle proliferation and fibrosis (e.g., miR‐130/301, miR‐21), as well as protective regulators of endothelial integrity and angiogenic capacity (e.g., miR‐126), underscoring how imbalance in miRNA networks may drive disease progression. Notably, several of these miRNAs influence both pulmonary vascular structure and myocardial stiffness, highlighting shared regulatory pathways that couple lung vascular dysfunction to cardiac remodeling.

**TABLE 1:** MicroRNAs involved in pulmonary vascular remodeling, endothelial and mitochondrial dysfunction, inflammation, and fibrosis relevant to PAH, HFpEF, and related conditions.

miRNA	Primary function	Pathophysiologic impact in PH‐HFpEF	Key references
miR‐130/131	Promotes smooth muscle cell proliferation and ECM reorganization via YAP/TAZ	Enhances pulmonary vascular stiffening and resistance, contributing to elevated filling pressures during exercise and impaired cardiopulmonary coupling	Bertero et al. (2014) and Brock et al. (2015)
miR‐210	Mediates hypoxia‐driven metabolic reprogramming, shifting cellular energetics toward glycolysis	Exacerbates energetic inefficiency and oxidative stress, limiting diastolic reserve and contributing to exercise intolerance	Guan et al. (2019) and Ahmed et al. (2024)
miR‐126	Preserves endothelial integrity and angiogenic capacity within the microvasculature	Loss of this signaling axis promotes microvascular rarefaction and endothelial dysfunction, central to reduced NO‐cGMP signaling	Wang et al. (2008) and Guenther and Schrepfer (2016)
miR‐21	Drives stress‐induced fibrotic signaling through activation of fibroblasts and smooth muscle cells	Accelerates interstitial fibrosis and myocardial stiffening, elevating LV filling pressures and impairing relaxation	Yamada et al. (2013)
miR‐29	Restrains ECM accumulation by suppressing collagen gene expression	Downregulation permits excess collagen deposition, increasing passive myocardial stiffness and worsening diastolic dysfunction	Cushing et al. (2011)
miR‐155	Amplifies immune and inflammatory signaling, particularly through macrophage activation	Sustains chronic inflammation and endothelial injury, promoting microvascular dysfunction and fibrotic remodeling	Shen et al. (2020) and Yu et al. (2022)

A key experimental advancement in the PH field was the demonstration that EVs can function as active mediators of disease, rather than passive byproducts of vascular injury. Using a monocrotaline (MCT) mouse model of PAH, Aliotta et al. isolated circulating EVs from diseased animals and showed that systemic transfer of these EVs to healthy recipients was sufficient to induce pulmonary hypertensive features, including vascular remodeling and hemodynamic changes. This finding provided compelling experimental evidence that EVs can convey a transferable pathogenic signal capable of reprogramming recipient tissues, establishing causality rather than simple association (Aliotta et al. 2013). Subsequent work by the same group further refined this concept by demonstrating that the exosome‐enriched fraction of EVs from MCT‐PAH mice was primarily responsible for inducing pulmonary hypertensive changes, while exosomes derived from healthy mesenchymal stem cells could prevent or reverse disease in the same model (Aliotta et al. 2016). These experiments were notable not only for implicating EVs as biologically active disease vectors, but also for revealing that EV subtype and cellular origin critically determine functional effects, underscoring the necessity of precise EV characterization for both mechanistic studies and therapeutic development.

A critical question is which molecular signals packaged within EVs are most relevant to pulmonary vascular remodeling in PH‐HFpEF. Among the best candidates are regulatory miRNAs that govern cellular proliferation, extracellular matrix remodeling, and vascular stiffening, processes increasingly recognized as central to pulmonary vascular pathology in HFpEF. Studies by Bertero et al. identified the miR‐130/301 family as a regulator of PH, demonstrating its upregulation in diseased pulmonary vessels and circulating plasma across experimental models and human cohorts (Bertero et al. 2014). A subsequent mechanistic study revealed that increased extracellular matrix stiffness activates YAP/TAZ signaling in pulmonary vascular cells, which in turn induces miR‐130/301 expression, establishing a feed‐forward loop that amplifies vascular remodeling (Bertero et al. 2015). Importantly, while this investigation did not directly examine EV‐mediated transfer in PH‐HFpEF or RV remodeling, it provides experimentally validated molecular cargo candidates and mechanistic pathways that are highly relevant to EV biology. These findings therefore offer a rational foundation for future PH‐HFpEF studies aimed at determining whether miRNA‐laden EVs participate in coordinating pulmonary vascular stiffening and right ventricular load, particularly in patients with combined pre‐ and post‐capillary disease.

Given the molecular signaling pathways discussed above, it is useful to delineate the distinct conceptual role of EVs. Unlike soluble mediators, EVs transmit packaged, stable, and specific signals across cells, linking pulmonary vascular communication to systemic and RV responses. One study demonstrated that circulating endothelial‐ and platelet‐derived microparticles are elevated in PH and correlate with disease severity, supporting their utility as biomarkers of vascular injury and dysfunction (Amabile et al. 2008). However, in PH‐HFpEF, the central unanswered question is not whether EVs are present, but whether EV signatures can distinguish clinically meaningful phenotypes, such as isolated post‐capillary versus combined pre‐/post‐capillary disease. Preclinical studies using mesenchymal stem cell‐derived EVs in Sugen‐hypoxia models demonstrate that EVs can actively modulate pulmonary vascular remodeling and RV hypertrophy, establishing biological plausibility for EV‐directed interventions (Klinger et al. 2020). Nevertheless, these findings cannot yet be extrapolated to PH‐HFpEF, where chronic left‐sided pressure overload, systemic metabolic inflammation, and advanced age are likely to alter EV composition, cellular targets, and therapeutic responsiveness.

Thus, the available evidence supports EVs as plausible mediators linking pulmonary vascular stress to systemic HFpEF pathophysiology. However, realizing their full mechanistic and translational potential in PH‐HFpEF will require disease‐specific studies that define EV source, cargo, and function, alongside standardized phenotyping approaches and integrative analyses combining EV profiling with invasive hemodynamics and exercise testing to establish relevance to pulmonary vascular reserve, RV‐PA coupling, and clinical outcomes.

## 4 Emerging Tools to Study Heart–Lung Interactions in PH‐HFpEF

Advances in animal models, imaging technologies, hemodynamic analyses, and computer model simulations have substantially expanded the ability to interrogate cardiopulmonary interactions across molecular, cellular, and organ‐level scales. These approaches are particularly important for PH‐HFpEF, a condition in which pulmonary vascular pathology and right ventricular mechanics interact dynamically to shape disease progression. Modern tools now permit simultaneous assessment of pulmonary vascular structure and function, RV mechanics, and interorgan signaling, enabling mechanistic insight into how lung and heart pathology evolve in parallel and give feedback to one another.

### 4.1 Animal Models for Mechanistic Discovery

Animal models remain indispensable for establishing causality and defining temporal relationships between pulmonary vascular remodeling and RV adaptation. Traditional PAH models, including Sugen‐hypoxia, chronic hypoxia, monocrotaline exposure, and pulmonary artery banding, have been instrumental in delineating the consequences of endothelial injury, inflammation, or mechanical afterload on RV remodeling (Wu et al. 2022). In particular, the Sugen‐hypoxia model recapitulates severe precapillary PH with vascular occlusion and RV hypertrophy, making it well suited for studying lung‐to‐heart signaling under extreme vascular stress (Vitali et al. 2014). However, PH‐HFpEF requires models that integrate cardiac, metabolic, and pulmonary insults rather than isolated precapillary disease. Recent small and large animal studies of HFpEF, including those incorporating PH, highlight the need for multi‐hit models that combine metabolic stress, pressure overload, aging, and vascular dysfunction to more accurately reproduce PH‐HFpEF pathobiology (Valero‐Muñoz et al. 2017; Jasińska‐Stroschein 2024).

In recent years, preclinical models of PH‐HFpEF have begun to provide important mechanistic insights into how metabolic stress, inflammation, and vascular dysfunction interact to drive disease progression. Work from Hemnes and colleagues has highlighted the role of inflammation and immune dysregulation in promoting pulmonary vascular remodeling and RV dysfunction in HFpEF‐associated PH, reinforcing the concept that PH‐HFpEF is not solely a hemodynamic disorder but also a biologically active, system‐wide disease (Agrawal et al. 2023). Complementary studies from Kuebler and colleagues have demonstrated that pulmonary vascular stiffening and endothelial barrier dysfunction contribute to altered cardiopulmonary mechanics, providing a mechanistic link between vascular remodeling and increased RV afterload (Liu, Nambiar Veetil, et al. 2022; Jaeschke et al. 2025). In parallel, Lai and colleagues have developed and characterized multi‐hit animal models, including high‐fat diet combined with NO synthase inhibition in mice and Sugen‐based models in obese ZSF1 rats, which recapitulate key features of PH‐HFpEF such as metabolic dysfunction, pulmonary vascular remodeling, and maladaptive RV responses (Jheng et al. 2023; Wang et al. 2019). Together, these studies advance the field by linking molecular and cellular signaling to integrative cardiopulmonary physiology while establishing more disease‐relevant platforms for therapeutic discovery.

### 4.2 Advanced Imaging of Cardiopulmonary Dynamics

Imaging technologies have evolved to allow noninvasive, high‐resolution assessment of cardiopulmonary structure and function in both preclinical and clinical settings. Echocardiography remains a gold standard tool, enabling rapid evaluation of RV size and systolic function. RV free‐wall strain measured by speckle‐tracking echocardiography is highly sensitive to early RV‐PA uncoupling and provides strong prognostic information in PH‐HFpEF (Melenovsky et al. 2014; Gorter et al. 2016; Sachdev et al. 2011). Four‐dimensional flow MRI has emerged as a powerful complementary modality, providing spatially resolved measurements of pulmonary artery flow patterns, regional stiffness, and wall shear stress (Barker et al. 2014). These parameters capture aspects of pulsatile afterload and RV kinetic energy that are poorly reflected by resting pressure measurements alone (Obokata, Kane, et al. 2017). When combined with MRI‐based RV volumetry and tissue characterization, advanced imaging offers an integrated, noninvasive lens into heart–lung interactions that bridges mechanistic animal studies and human disease.

### 4.3 Hemodynamic Monitoring and Pressure‐Volume Analysis

Invasive hemodynamic assessment remains a critical tool for quantifying RV‐PA coupling and pulmonary vascular function (Sanz et al. 2012). High‐quality pressure measurements, conductance catheter‐based pressure‐volume loop analysis, and exercise hemodynamics allow precise determination of RV contractility, afterload, diastolic stiffness, and energetic efficiency. These measures are particularly relevant in PH‐HFpEF, where abnormalities in compliance, pulsatile load, and reserve often emerge during stress rather than at rest. Wave intensity and wave separation analyses further enable decomposition of RV afterload into steady and pulsatile components, revealing how pulmonary vascular stiffening and early wave reflection increase RV wall stress (Lechuga et al. 2025b; Yim et al. 2024). Continuous hemodynamic monitoring in large animal models allows investigators to track how the RV adapts over time and to identify when specific mechanical or molecular stressors trigger the transition from compensation to decompensation (Ukita et al. 2021).

### 4.4 Computational and Systems Modeling of RV‐PA Interaction

Computational modeling provides a unifying framework to integrate experimental and clinical data across scales. Lumped‐parameter (Windkessel) models, finite‐element simulations of RV mechanics, and fluid–structure interaction models of the pulmonary vasculature enable quantitative prediction of how changes in pulmonary vascular resistance, compliance, geometry, and wave propagation alter RV afterload and workload (Kachabi et al. 2023; Altieri Correa et al. 2025; Kheyfets et al. 2016). These models can be paired with machine learning and parameter inference approaches to capture nonlinear interactions, simulate disease trajectories, and test therapeutic interventions in silico (Cai et al. 2025). Such integrative modeling is especially valuable in PH‐HFpEF, where venous congestion, pulmonary vascular remodeling, and RV adaptation interact to produce heterogeneous phenotypes that complicate traditional reductionist approaches.

### 4.5 Extracellular Vesicle Tracking and Multi‐Omics Integration

Recent technological advances now permit direct interrogation of molecular communication between lung and heart. Fluorescent and radiolabeled EV tracking strategies enable visualization of vesicle biodistribution and cellular uptake in vivo, clarifying how EV‐mediated signals propagate across the cardiopulmonary axis (Liu, Huang, et al. 2022). In parallel, single‐cell RNA sequencing, spatial transcriptomics, proteomics, metabolomics, and miRNA profiling provide high‐resolution maps of the molecular networks governing pulmonary vascular and RV remodeling (Saygin et al. 2020). Integrating multi‐omics data with imaging and hemodynamic measurements enables identification of pathway‐specific modifiers, such as inflammatory signaling, hypoxia‐responsive programs, estrogen receptor signaling, and metabolic dysfunction, that uniquely shape cardiopulmonary adaptation in PH‐HFpEF. Together, these approaches offer powerful tools for defining disease mechanisms, discovering biomarkers, and identifying therapeutic targets.

## 5 Emerging Therapies for PH‐HFpEF

With multiple randomized trials of pulmonary vasodilator therapies failing to demonstrate benefit in PH‐HFpEF, including phosphodiesterase‐5 inhibitors and endothelin receptor antagonists (Dachs et al. 2022; Sardar 2022; Hoeper et al. 2024), clinical management has largely remained focused on established HFpEF therapies such as sodium‐glucose cotransporter‐2 inhibitors (SGLT2i), glucagon‐like peptide‐1 receptor agonists (GLP1a), and mineralocorticoid receptor antagonists (MRA) (Shah 2026). Nevertheless, there remains an unmet need for PH‐HFpEF specific treatments, and several emerging therapies are currently undergoing evaluation in human clinical trials. Endovascular pulmonary artery denervation has demonstrated hemodynamic benefit in early studies (Rothman et al. 2019) and is now being investigated using both ultrasound‐based (NCT06052072) and radiofrequency‐based catheter systems (NCT05824923). Levosimendan has also shown promise in PH‐HFpEF, with intravenous administration improving hemodynamics through combined inotropic and vasodilatory effects mediated by potassium channel activation, myofilament calcium sensitization, and phosphodiesterase‐3 inhibition (Burkhoff et al. 2021), leading to an ongoing clinical trial of oral levosimendan in PH‐HFpEF patients (NCT05983250). In parallel, sotatercept represents a first‐in‐class therapeutic approach targeting aberrant cell proliferation via modulation of activin/TGF‐β signaling, distinct from traditional PAH vasodilators (Nagaraj et al. 2025). Following its demonstrated efficacy in reversing pulmonary vascular remodeling in PAH (Humbert et al. 2021), sotatercept has recently been evaluated in patients with PH due to heart failure in the CADENCE trial. Results from the CADENCE trial indicate that activin signaling inhibition improves pulmonary vascular and cardiac hemodynamics in this population, providing early clinical evidence that targeting proliferative and remodeling pathways may be beneficial in PH‐HFpEF (Gomberg‐Maitland et al. 2026). These findings contrast with prior trials of pulmonary vasodilators in HFpEF and support a shift toward therapies that address underlying vascular and myocardial remodeling. Finally, the Aria pulmonary endovascular device, which improves pulmonary arterial compliance through implantation of a compressible balloon within the main pulmonary artery, has demonstrated a ~20% increase in compliance in PAH patients (Gerges et al. 2024). While current trials of this device remain focused on PAH (NCT04555161), this biomechanical strategy may hold promise for selected PH‐HFpEF populations.

## 6 Knowledge Gaps and Future Directions

Despite growing recognition that PH‐HFpEF represents a disorder of maladaptive heart–lung interactions, major gaps remain in our mechanistic understanding and clinical characterization of this syndrome. Much of the current framework for pulmonary vascular disease and RV adaptation has been derived from precapillary PAH, yet PH‐HFpEF arises in a distinct biological context defined by chronic venous pressure overload, systemic inflammation, metabolic dysfunction, and aging. A central challenge for the field is therefore to delineate which aspects of pulmonary vascular remodeling, RV‐PA coupling, and molecular signaling are shared across forms of PH and which are unique to PH‐HFpEF to create disease‐specific models and therapeutic strategies.

One critical gap is the limited availability of experimental models that recapitulate PH‐HFpEF biology. While hypoxia‐ and toxin‐based models, such as MCT exposure, have been valuable for defining pulmonary vascular remodeling and RV failure, they do not capture the combined influence of left‐sided pressure overload, metabolic stress, and systemic inflammation that characterizes PH‐HFpEF. Emerging multi‐hit models incorporating pressure overload, metabolic dysfunction, and aging offer promise, but remain underutilized and incompletely characterized (Meng et al. 2017; Liu, Yan, et al. 2022). Future studies must leverage such models to interrogate temporal relationships between pulmonary venous hypertension, pulmonary arterial stiffening, and RV adaptation, including sex‐specific differences that are highly relevant given the epidemiology of HFpEF.

A second major gap lies in the integration of molecular and cellular signaling with physiological phenotyping. Although pathways involving NO‐cGMP signaling, ET‐1, hypoxia‐responsive transcriptional programs, and sex hormone signaling have been implicated in PH‐HFpEF, these mechanisms are rarely studied in parallel with direct measures of pulmonary vascular reserve, pulsatile load, or RV‐PA coupling. Bridging this divide will require experimental and clinical studies that pair molecular profiling with invasive hemodynamics and exercise testing, allowing signaling abnormalities to be interpreted in the context of dynamic cardiopulmonary function rather than resting pressure measurements alone.

EVs represent a compelling but underdeveloped area of investigation. While EVs have been shown to mediate pulmonary vascular remodeling and RV adaptation in other forms of PH, their role in PH‐HFpEF remains largely unexplored. Key unanswered questions include the cellular origin of circulating EVs in PH‐HFpEF, how EV cargo is shaped by venous congestion and metabolic inflammation, and whether EV signatures can distinguish clinically meaningful phenotypes or predict progression to RV dysfunction. Addressing these questions will require standardized EV isolation strategies, as well as integration of EV profiling with physiologic and imaging‐based measures of cardiopulmonary coupling.

From a clinical perspective, phenotyping remains a major limitation. Current diagnostic paradigms rely heavily on resting hemodynamic indices that incompletely capture the dynamic nature of pulmonary vascular dysfunction in PH‐HFpEF. Advanced imaging, exercise hemodynamics, and computational modeling provide opportunities to redefine PH‐HFpEF based on functional reserve, pulsatile load, and RV energetic efficiency rather than static thresholds alone. Future work should focus on identifying combinations of physiological, molecular, and imaging markers that can stratify risk, guide therapy, and improve clinical trial design.

In summary, PH‐HFpEF represents a complex, multiscale disorder in which mechanical forces and molecular signaling intersect across the cardiopulmonary unit. Progress in this field will depend on approaches that integrate disease‐relevant models, advanced physiological assessment, and systems‐level molecular profiling to capture interactions that span molecular, cellular, and organ‐level scales. By explicitly embracing heart–lung interactions as the central organizing principle, future studies have the potential to transform both mechanistic understanding and clinical management of this increasingly prevalent and deadly condition.

## Funding

This study was supported by the Edwards Lifesciences Foundation Fellowship (P.P.), the National Center for Advancing Translational Sciences, Grant/Award Number: KL2TR002374‐07 (F.R.), American Heart Association, Grant/Award Number: 23CDA1057697 (F.R.), NIH R01HL154624 (N.C.C.), and NIH R01HL147590 (N.C.C.). The content is solely the responsibility of the authors and does not necessarily represent the official views of the NIH.

## Conflicts of Interest

N.C.C. has consulted for Aria CV and Gradient Denervation Technologies. F.R. oversees a clinical trial for Gradient Denervation Technologies.

## Data Availability

The authors have nothing to report.
